# Training Healthcare Assistants for School-Based Care of Children Receiving Paediatric Palliative Care: A Post-Training Evaluation

**DOI:** 10.3390/children13010153

**Published:** 2026-01-22

**Authors:** Anna Santini, Anna Marinetto, Enrica Grigolon, Alessandra Fasson, Mirella Schiavon, Igor D’angelo, Nicoletta Moro, Barbara Roverato, Pierina Lazzarin, Franca Benini

**Affiliations:** 1 Pediatric Palliative Care, Pain Service, Department of Women’s and Children’s Health, University Hospital of Padua, 35128 Padua, Italy; anna.santini@aopd.veneto.it (A.S.); anna.marinetto@aopd.veneto.it (A.M.); enrica.grigolon@aopd.veneto.it (E.G.); alessandra.fasson@aopd.veneto.it (A.F.); mirella.schiavon@aopd.veneto.it (M.S.); igor.dangelo@aopd.veneto.it (I.D.); nicoletta.moro@aopd.veneto.it (N.M.); barbara.roverato@aopd.veneto.it (B.R.); pierina.lazzarin@unipd.it (P.L.); 2 Department of Women’s and Children’s Health, University of Padua, 35128 Padua, Italy

**Keywords:** paediatric palliative care, school care assistants, school inclusion, simulation training, family partnership

## Abstract

**Highlights:**

**What are the main findings?**

- Short, simulation-based training was associated with high self-reported perceived confidence in routine and emergency tasks in school settings immediately after the session.
- Training that includes child-specific scenarios and family-partnership elements was perceived as helpful for supporting safer school inclusion for children with complex needs.

**What are the implications of the main findings?**

- Modular programs that combine a common core with individualized modules may help address the heterogeneous experiences and needs of staff.
- Regular practical refreshers and objective skills checks are recommended to sustain readiness for low-frequency/high-impact events and to strengthen trust with families.

**Abstract:**

**Background/Objectives**: Children in paediatric palliative care often face school attendance barriers due to complex health needs. This study describes post-training perceptions of a training program by a pediatric hospice team to prepare school care assistants to safely include children with complex conditions, focusing on procedural skills, knowledge of the child, and family partnership. **Methods**: Care assistants who completed a structured course at the Paediatric Palliative Care Centre, University Hospital of Padua (2023–2024), were surveyed immediately after training. The program combined classroom instruction with hands-on simulation using high-fidelity mannequins and standard devices, including suction, pulse oximetry, ventilation, enteral feeding, and tracheostomy care. It also covered modules on urgent and emergency management, as well as family communication. An anonymous online questionnaire gathered socio-demographic data, prior training, clinical tasks performed, self-efficacy levels, and open-ended feedback. Quantitative results were analyzed descriptively, while qualitative comments were subjected to thematic analysis. **Results**: Of 130 invited assistants, 105 participated (81%). Participants reported strong perceived confidence: 85% selected the upper end of the 5-point scale (“very” or “extremely”) for routine-management ability, and 60% selected these same response options for emergency-management ability. In the most severe events recalled, 60.5% of incidents were resolved autonomously, 7.6% involved contacting emergency services, and 3.8% involved community or hospice nurses. Seventy-five percent judged the course comprehensive; thematic analysis of 102 comments identified satisfaction, requests for regular refreshers, stronger practical components, and requests for targeted topics. **Conclusions**: Immediately after the session, participants tended to select the upper end of the self-assurance item for both routine and emergency tasks. Combining core emergency procedures with personalized, child-specific modules and family-partnership training may support safety, trust, and inclusion. Regular refreshers and skills checks are advised.

## 1. Introduction

Paediatric palliative care (PPC) encompasses the provision of care for children suffering from incurable diseases that either restrict or pose a threat to their lives. Children and their families frequently contend with a chronic condition that presents intricate clinical, psychosocial, and ethical challenges, often accompanied by an uncertain prognosis. These conditions are characterized by high medical complexity, the utilization of life-support devices, and issues that transcend purely medical concerns [1].

Indeed, families with children in specialized palliative care face daily caregiving challenges and unpredictable prognoses. However, evidence indicates that these families need support, effective collaboration among healthcare providers, clear communication, and training to improve their perceptions of quality of life [2,3].

Furthermore, in addition to addressing clinical and caregiving requirements, services must acknowledge that children have the right to develop social skills, including the right to participate in play, enjoy leisure activities, attend educational institutions, and establish peer relationships. These rights ought to be consistently supported and tailored to align with the child’s developmental stage and physical condition [4,5,6,7].

In particular, the school serves as a vital environment for developing children’s social skills and their relationships with peers and teachers.

Consequently, fostering coordination and communication between healthcare professionals and educational institutions is imperative to address care needs and formulate effective treatment strategies. Such collaboration can lead to improved school attendance, increased engagement, active participation, and enhanced academic achievement among students [8]. The American Academy of Pediatrics advocates for communication between healthcare practitioners and schools, emphasizing that all physicians should recognize the importance of comprehensive, coordinated teamwork among medical centers, schools, and the child’s family. Healthcare providers are encouraged to support their patients and bolster local school health initiatives by collaborating closely with school health services teams [9]. This approach, however, presents additional challenges for children with special and complex health needs who require supplementary services to facilitate their success in educational environments [10]. In Italy, children are typically supported by nurses; alternatively, support may be provided by educators, teachers, Healthcare Assistants, or even parents [11].

We organized a training program for Healthcare Assistants (HCAs), conducted by our Paediatric Palliative Care Center. This study intends to assess the perceived efficacy of the HCA training model.

In the Italian system, Healthcare Assistants (HCAs) hold a professional qualification, obtained through a post-secondary programme of at least 1000 h, divided between theoretical coursework and supervised clinical practice. Their training prepares them to provide basic care, support daily activities, and perform simple delegated procedures within educational and home-care settings. HCAs trained through this programme work predominantly in public schools, while a smaller proportion operate in special education settings, depending on the child’s functional profile and educational plan.

## 2. Materials and Methods

The experimental training project for HCAs was conducted and managed by specialized nursing staff to ensure safe school attendance for children and adolescents (ages 3–18) enrolled in the PPC program. Each year, approximately 100 HCAs are trained to support daily activities—including feeding, respiratory care, and hygiene—and to perform essential health procedures that keep the child safe and healthy in school. The training follows a standardized model that includes structured theoretical lessons, high-fidelity mannequin-based practical simulations, and collaborative support from local health and social services, school staff, and teachers.

The intervention consisted of a half-day training session (approximately 4 h), comprising ~2 h of theoretical instruction and ~2 h of practical simulation. Training was delivered by specialized pediatric palliative care nurses and a clinical psychologist, focusing on specific child-related emotional or behavioral characteristics, with an instructor-to-participant ratio of approximately 1:3 to ensure close supervision during simulations. All simulation scenarios were standardized using predefined scripts, equipment checklists, and expected actions, ensuring consistent delivery across sessions. After each scenario, a brief debriefing was conducted, encouraging participants to reflect on their actions, clarify doubts, and consolidate key procedural steps. The training targeted core competencies, including safe management of daily care procedures, early identification of clinical changes, and first-response actions in urgent situations.

The training is customized to each child’s specific needs and combines theoretical lessons with realistic, scenario-based practical exercises. This approach aims to enhance child safety at school, reduce the caregiving burden on families, and make school inclusion a core part of the individualized care plan.

The study used a descriptive observational design. Healthcare Assistants (HCAs) who had completed the PPC Centre’s structured training program during 2023–2024 were invited to participate voluntarily and anonymously in an online survey. The questionnaire was distributed through the coordinators of the cooperatives collaborating with the Centre and sent to 130 eligible HCAs. A total of 105 participants responded, yielding an overall response rate of 81%.

The survey, conducted from 19 February to 14 March 2025, utilizing Google Forms, comprised 19 questions segmented into five sections: socio-demographic data (10 questions), skills related to managing daily and emergency care procedures (5 questions), perceived confidence and preparedness (single-item indicators rather than a psychometric self-efficacy scale) (1 question), the relationship with PPC staff (3 questions), and a section dedicated to free comments (1 question). The complete questionnaire, including the exact wording of items and response options, is provided in the Supplementary Materials.

The instrument included dichotomous items (yes/no; yes/no/other), multiple-choice questions, multiple-response checklists, and 5-point Likert scales ranging from “not at all” to “extremely.” To ensure transparency and fidelity to the original response format, no derived categories (e.g., high/medium/low) were used in the analysis; instead, Likert responses were reported directly, with specific attention to selections at the upper end of the scale.

Quantitative data were analyzed using simple descriptive statistics (frequencies and percentages). No inferential analyses or subgroup comparisons were performed, as the study was designed as a preliminary descriptive evaluation. Because each domain of perceived confidence and preparedness was assessed with single-item measures, internal consistency indices (e.g., Cronbach’s alpha) are not applicable. The questionnaire underwent internal review by the clinical and educational team to ensure clarity and relevance, but no formal piloting was conducted.

Participation in the survey was voluntary and anonymous. Completing the questionnaire was considered informed consent. Data management adhered to current privacy and personal data protection laws (Regulation EU 2016/679—General Data Protection Regulation GDPR). The data, collected in an anonymous, aggregated form, were analyzed using descriptive statistics in Jamovi (version 2.6.26.0) [12,13,14].

Free-text responses were analysed using a simplified qualitative content-analysis approach. Two researchers independently reviewed all open-ended answers, assigned descriptive codes to recurring ideas, and then compared their coding to reach consensus. Codes were subsequently grouped into broader descriptive categories, allowing us to summarise the main patterns emerging from the freeform items. Given the brevity and non-narrative nature of the responses, the analysis remained descriptive rather than interpretative.

### 2.1. Training Model Description

The training was developed to address a concrete operational need: enabling school personnel to safely support children with medical devices during daily school activities. Its structure is deliberately concise and practice-oriented, focusing on essential procedures and on building the confidence required to manage both routine care and potential emergencies. Although the intervention originated in practical considerations rather than in a formal theoretical framework, its design was intentionally informed by well-established principles in clinical education.

The progression from basic knowledge to applied competence reflects the logic of the Kirkpatrick model—particularly Levels 2 and 3, which emphasize learning outcomes and the transfer of skills to real-world behavior [15,16]. Similarly, the use of demonstration, guided practice, and feedback aligns with Social Cognitive Theory, in which observational learning and self-efficacy are central mechanisms for developing competent performance [17,18].

From operational and pedagogical perspectives, high-fidelity simulation was selected as the core instructional method because it enables rapid skill acquisition and builds learners’ confidence in managing low-frequency, high-impact events. Scenario-based learning, deliberate practice, and structured debriefing—well-established strategies in simulation-based education—were intentionally incorporated to support safe, reliable responses in emergency situations [19,20].

The Specialist Paediatric Palliative Care Centre organizes training for all HCAs responsible for children’s care within its remit, to promote safe and consistent school attendance. Before the training, PPC trainers collect detailed clinical and care information on paediatric patients by engaging directly with healthcare professionals such as territorial nurses and family doctors, consulting families, and reviewing clinical records. This preparatory step ensures that the training is customized to meet each patient’s unique needs.

Training occurs in a dedicated classroom fitted with high-fidelity mannequins and specialized medical devices to replicate essential care procedures. The curriculum, aligned with the child’s care plan, focuses on three key areas: technical assistive procedures, medical device operation, and emergency response.

### 2.2. Technical-Assistive Procedures

- Aspiration of oro-nasal secretions.
- Administration of enteral feeding via pump and/or syringe.
- Basic technical handling of gastrostomy tube, nasogastric tube (NG tube).
- Management of epileptic seizures and administration of prescribed emergency medications.
- Tracheostomy care, including suction and emergency cannula replacement.

### 2.3. Use of Medical Devices

- Self-inflating bag (Ambu) for ventilation support.
- Basic operation, start-up, and alarm management of home mechanical ventilators.
- Use and maintenance of suction machines.
- Using a pulse oximeter to monitor vital signs.

### 2.4. Urgent/Emergency Procedures

- Activation of emergency medical services (Italian EMS number 118) according to a case-specific flowchart.
- Recognition and initial management of urgent or emergency scenarios.
- Paediatric cardiopulmonary resuscitation (CPR) in children with and without tracheostomy.
- Placement in the recovery position.
- Airway obstruction removal (Heimlich manoeuvre).
- Using cough assist devices in emergencies.

Intervention areas are chosen based on each child’s needs, totaling 4 h of focused training. After completing both theoretical and practical parts, HCAs engage in a supervised shadowing phase with the child’s caregivers or home nursing staff. Usually, HCAs need to perform each required skill around three times under supervision to be deemed competent. The duration of this phase varies and is decided through agreements with the family and local services. Following this, HCAs take on independent responsibilities.

After the supervised phase, HCAs perform routine care tasks, monitoring, and device-related activities specified in each child’s personalized care plan. Their work isn’t without oversight; PPC nurses and pediatricians develop a flowchart for managing urgent issues or emergencies for each child. Post-training, the PPC network offers continuous clinical support, with staff available 24/7, and maintains regular contact with the child’s healthcare team to track the child’s needs at school. In addition to PPC supervision, local services—such as home care teams and the child’s family pediatrician—also supervise the HCA’s activities, provide guidance, and intervene as needed, based on their clinical judgment. This approach ensures that HCAs perform their duties within a regulated, well-supported framework, promoting safety and consistency in school settings.

**Figure 1:**
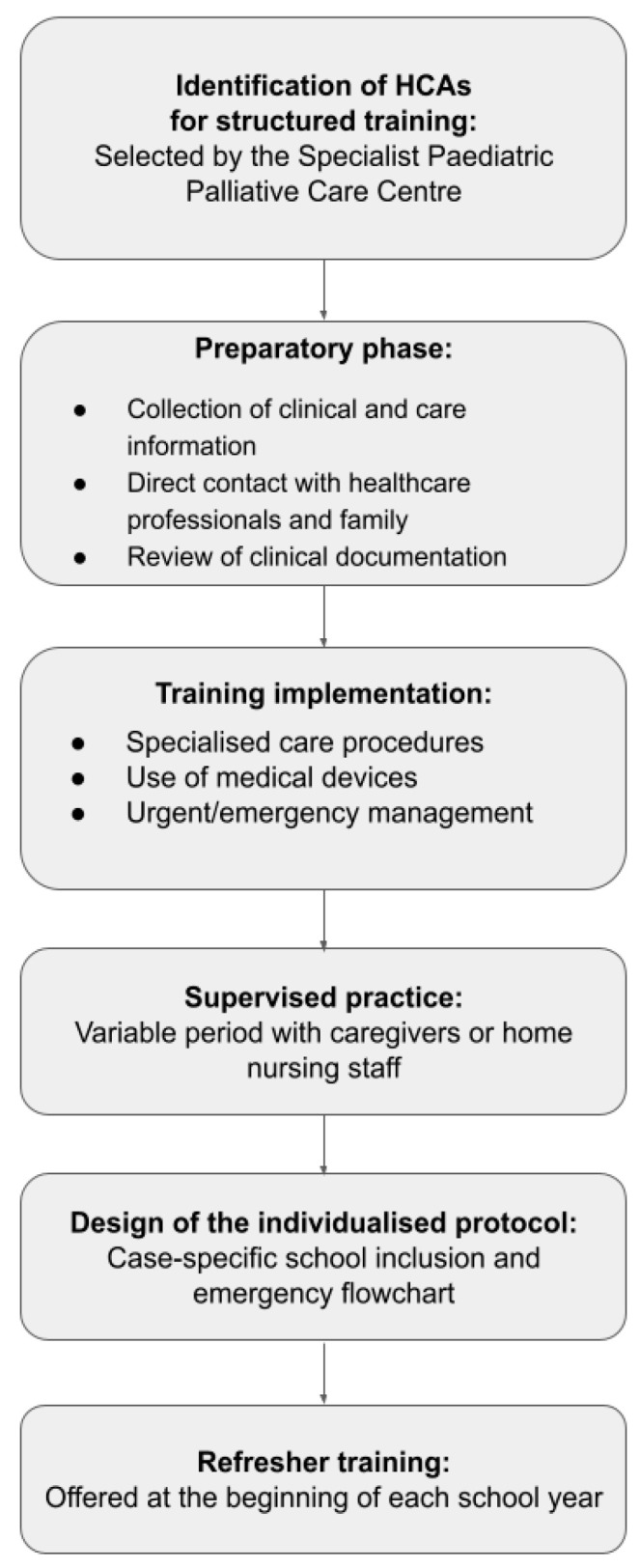
Operational Process for Deploying HCA Training.

To maintain and update competencies, HCAs can attend a refresher training session at the start of each school year. A schematic overview of the training process is shown in Figure 1.

## 3. Results

### 3.1. Cohort Characteristics and Prior School Experience

The cohort included 105 HCAs (102 female, 97.1%); mean age was 46.4 years (SD 9.25; range 24–69; N = 101). Most held a 5-year upper secondary diploma (73; 70.9%), while 24 (23.3%) had a lower secondary qualification (in Italy, the lower secondary qualification is typically obtained at around age 14, while the upper secondary qualification is usually awarded at around age 19), and 5 (4.9%) had a university degree. Work experience in schools was most frequently 2–4 years (45; 42.9%); 36.2% reported >8 years, and 61.5% had cared for one PPC-assigned child. Regarding prior training, 59.0% completed BLS/PBLS (Basic Life Support/Pediatric Basic Life Support), 20.0% attended other PPC-related courses, and 48.6% reported coaching/contextualization with specialized staff (see Supplementary Materials for: Tables S1–S3, Figure S1).

### 3.2. Clinical Tasks and Post-Training Event Frequency

Reported clinical needs (105 cases; total responses = 338) were dominated by nasal/oropharyngeal suctioning (97 cases; 97.0% of cases) and gastrostomy care (63 cases; 63.0%) (Table 1).

**Figure 2:**
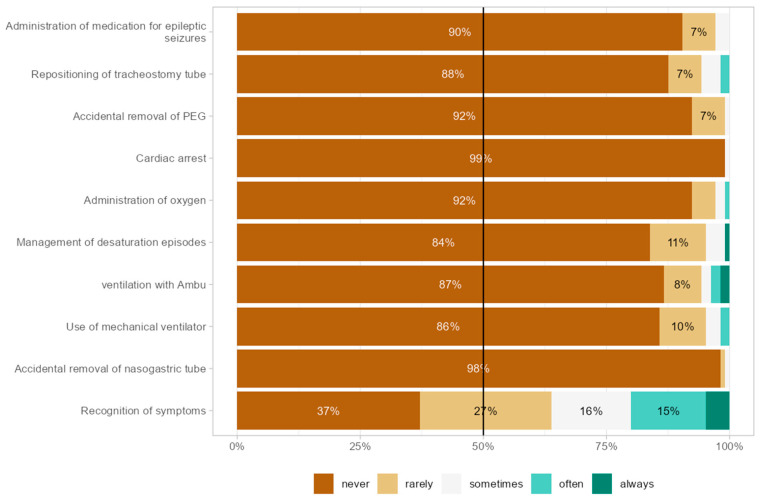
Frequency of tasks and procedures performed at school.

Other frequently reported needs included medication administration (35 cases; 35.0%) and enteral feeding management with pump/syringe (29 cases; 29.0%). Aggregated post-training frequencies of tasks and procedures performed at school (Figure 2) show recognition of symptoms (e.g., fever) as the most commonly performed task sometimes/often (31%), followed by oxygen administration (8%); tracheostomy cannula replacement, desaturation management, and Ambu ventilation were each reported sometimes/often by approximately 4–5%.

### 3.3. Severe/Urgent Events and Their Management

When asked to recall the most severe or urgent episode they had encountered (N = 70), HCAs most frequently reported severe desaturation events (12; 34.3%) and prolonged epileptic seizures requiring emergency medication (6; 17.1%). Unintentional displacement or repositioning of the tracheostomy cannula was also standard (6; 17.1%). Less frequent but clinically relevant events included accidental removal of the gastrostomy tube (5; 14.3%), aspiration-related episodes such as excessive mucus accumulation or suspected aspiration during feeding (2; 5.7%), meal-related emergencies (e.g., choking or difficulty managing food boluses) (2; 5.7%), and a single case of foreign body inhalation (1; 2.9%).

Regarding the management of these events (N = 70), most were handled autonomously by HCAs (26; 60.5%), while 29 episodes (27.6%) required contacting the child’s parents. External professional support was needed in a minority of cases: emergency medical services were activated in 8 events (7.6%), and territorial or PPC nursing staff intervened in 4 events (3.8%). These escalations occurred primarily during severe desaturation episodes, prolonged seizures, or unintentional tracheostomy cannula removal. The percentages reflect the management pathways chosen by HCAs rather than clinical outcomes, as the survey did not collect patient-level outcome data.

### 3.4. Post-Training Perceptions

**Figure 3:**
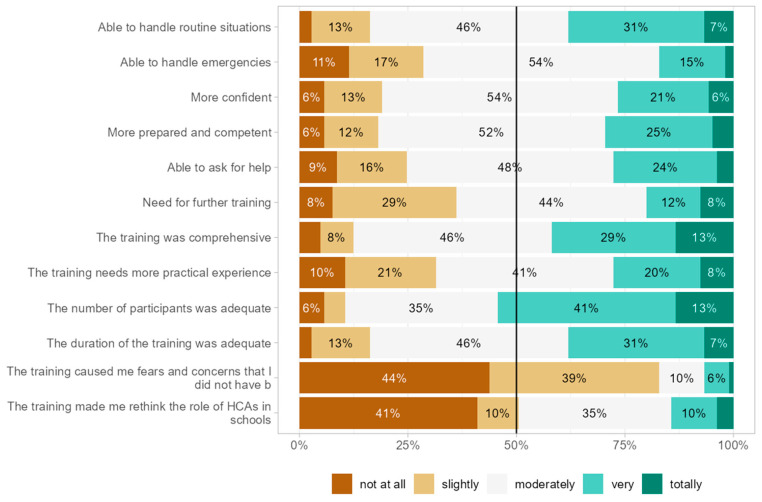
Individual perceptions after training.

Aggregated post-training perceptions were largely positive (Figure 3). 85% felt very or extremely able to manage routine situations, and 60% felt very or extremely able to handle emergencies. 70% reported perceived preparedness, 80% reported increased preparedness, and 75% rated the training’s completeness very highly. The majority (65%) reported little or no further need for specialised training, while 15% expressed a marked need for additional interventions; only 5% reported marked increases in fears/worries, and 20% reconsidered their HCA role.

Below is a summary of the 102 free comments collected, organized into five themes. For each theme, we report the number and percentage of comments, along with a brief explanation and short verbatim quotes selected to represent the most recurrent perspectives within each theme.

- Theme 1: Satisfaction and appreciation of the training (21 comments, 20.6%). Participants frequently expressed strong appreciation for the trainers’ clarity and professionalism. Several comments emphasized that the course was “apparent and reassuring” and that the trainers were “brave, concrete, and extremely competent”. Overall, the training was perceived as effective and well delivered.
- Theme 2: Periodic updates and training frequency (35 comments, 34.3%). A large proportion of participants requested regular refresher sessions to maintain confidence and prevent skill decay. Comments included statements such as “a refresher every six months would be very useful” and “it would be important to repeat the training at least once a year.” Many participants emphasized that continuous updates are essential when working with children with complex care needs.
- Theme 3: Strengthening practical training (11 comments, 10.8%). Participants often requested more hands-on practice, ideally in real-life contexts. Several noted that “practice directly with the child is the only way to learn” and that “more practical hours would help us feel more confident.” Some also preferred smaller groups to allow more individual practice.
- Theme 4: Specific training on targeted topics (9 comments, 8.8%). This theme includes requests for additional focused training on specific procedures, such as cardiopulmonary resuscitation, seizure management, gastrostomy care, and respiratory device use. Participants noted that “a refresher on CPR and machinery would be very useful” and that updates are needed as technologies and medications evolve.
- Theme 5: Other (26 comments, 25.5%). This theme includes comments that did not fit neatly into the previous categories, such as logistical suggestions (e.g., “a more accessible location would help”), requests for opportunities to share experiences with colleagues, and general expressions of gratitude (“the training was very useful; thank you for your availability and competence”).

## 4. Discussion

The diversity in age, years since HCA certification, and prior paediatric complex care experience among participants shows that a uniform training approach is insufficient. A fundamental core curriculum should be established to provide a safe baseline, focusing on vital emergency and monitoring skills, such as symptom recognition, oxygen delivery, airway support, and care for gastrostomy and tracheostomy. This core should be complemented with modular, competency-based extensions tailored to each HCA, taking into account their previous experience and the specific needs of the children they will care for serve. In our post-training evaluation, participants indicated increased perceived competence following brief, targeted sessions. This approach could be especially beneficial if incorporated into a structured pathway with scheduled refreshers to enhance medium-term retention [21].

Programme design must explicitly incorporate two relational dimensions. The first is a thorough understanding of each child’s unique situation, including their baseline clinical status, personalized care plan, and known deterioration triggers. This knowledge is vital for safe and prompt responses. Therefore, training should encompass structured handovers, case reviews, and scenario exercises based on actual patient profiles, ensuring that procedural skills are directly connected to person-centered understanding. Secondly, involving families is essential: parents entrust the care of complex, often vulnerable children to school staff, often feeling anxious and seeking transparent communication and shared responsibility. Incorporating relational modules during onboarding—using role-plays, simulated briefings, and co-developing contingency plans—can build trust and enhance the smoothness of transitions between home and school [22].

HCAs primarily focus on monitoring and recognizing symptoms, while performing advanced procedures is less common but crucial when needed. To balance the infrequent occurrence of these high-impact events with the need for readiness, programs should combine focused, high-quality practice for rare, high-impact situations with short, regular refreshers such as micro-learning or brief simulations, along with supervised in situ shadowing or mentoring. Objective evaluations, such as observed skills checks and scored scenarios, should supplement self-assessment methods to verify competence and facilitate personalized skill development. This approach aligns with evidence suggesting that structured retraining and practical reinforcement enhance skill retention [23].

This evaluation has some limitations: it relies on self-reported perceptions gathered immediately after training, which may be influenced by social desirability bias. It also lacks objective assessments, real-world observations, or follow-up on whether skills are retained over time. The sample was mostly female (97.1%), aligning with the local workforce but restricting the applicability of the findings to more diverse groups. As a post-training evaluation conducted at a single centre, the results should be interpreted with caution: they reflect perceived confidence rather than proven competence and cannot be generalized to other organisational models or long-term outcomes. Given that this was a preliminary, descriptive evaluation at a single centre, the study was neither intended nor sufficiently powered for inferential analyses or subgroup comparisons. Therefore, the results should be viewed as descriptive and not extended beyond this specific context. Nonetheless, the consistent quantitative patterns and detailed qualitative feedback—such as requests for refreshers, more practical elements, and customized modules—provide practical insights for ongoing improvements and future multicentre research.

## 5. Conclusions

In this post-training assessment, brief, targeted interventions correlated with higher self-reported readiness, as shown by participants choosing the top of the confidence scale for both routine monitoring and emergency care. To turn perceived improvements into enduring, quantifiable skills and fulfill families’ genuine expectations, future programs should adopt an integrated approach. This approach would feature a core curriculum covering essential safety procedures, personalized learning pathways tailored to prior experience and individual children’s needs, and relationship-focused training that fosters strong HCA–family collaboration. Integrating child-specific briefing practices, supervised shadowing within the child’s care setting, and communication rehearsals with parents from the beginning of the course may enhance both technical skills and relational trust. Regular objective assessments and scheduled refresher sessions could support ongoing competence in rare but critical events and promote safe escalation techniques. By merging clinical expertise with relational intimacy and structured family involvement, training can serve as a concrete step toward safer, more inclusive schooling for children with complex health needs, reassuring families and empowering professionals to act confidently and competently.

This study is a pilot, pragmatic evaluation of a PPC-led training approach. Larger, controlled trials with pre-/post-assessments, objective skills evaluations, and patient-centered outcomes are necessary to verify effectiveness, examine medium-term retention, and inform broader implementation across varied settings.

## Figures and Tables

**Table 1:** Types of children’s care needs (Number of cases: 105).

Clinical Needs	Counts	% of Responses	% of Cases
Gastrostomy care	63	18.6%	63.0%
Nasal/oropharyngeal suctioning	97	28.7%	97.0%
Medication administration	35	10.4%	35.0%
Management of enteral feeding with a pump/syringe	29	8.6%	29.0%
Use of a cough assist device in emergencies	27	8.0%	27.0%
Use of a pulse oximeter	26	7.7%	26.0%
Tracheostomy management	25	7.4%	25.0%
Ventilation with Ambu	23	6.8%	23.0%
Use of a home ventilator	9	2.7%	9.0%
Bladder catheterization	4	1.2%	4.0%
**Total**	**338**	**100.0%**	**-**

## Data Availability

The data presented in this study are available on request from the corresponding author due to privacy reasons.

## Supplementary Materials

The following supporting information can be downloaded at: https://www.mdpi.com/article/10.3390/children13010153/s1, Table S1: Cohort characteristics; Table S2: Duration of employment in schools; Table S3: Specialist training courses; Figure S1: Number of children in CPP who have been assigned to each HCA since they started working at the school.

## Abbreviations

The following abbreviations are used in this manuscript:

PPC	Paediatric Palliative Care
HCA	Healthcare Assistant
NG	Nasogastric Tube
CPR	Cardiopulmonary Resuscitation
BLS	Basic Life Support
PBLS	Paediatric Basic Life Support
