# Supplementary material for: Training Healthcare Assistants for School-Based Care of Children Receiving Paediatric Palliative Care: A Post-Training Evaluation
Source: Children (Basel). 2026 Jan 22;13(1):153. doi: 10.3390/children13010153 (PMC12839627; doi:10.3390/children13010153)
Supplement: Supplementary file 1 [file children-13-00153-s001.zip › children-4069541-supplementary.pdf]

Table S1 — Cohort characteristics

Variable	Level / Statistic	Frequency	% of Total	% Cumulative
**Gender**	Female	102	97.1%	97.1%
	Male	3	2.9%	100%
**Educational Qualification**	Lower secondary school	24	23.3%	23.3%
	Upper secondary school (3y program)	1	1.0%	24.3%
	Upper secondary school (5y program)	73	70.9%	95.1%
	University degree	5	4.9%	100.0%
**Age** (N = 101; missing = 4)	Mean	46.4		
	Median	48.0		
	Standard Deviation	9.25		
	Min.	24.0		
	Max.	69.0		
**Year of HCA graduation** (N = 104; missing = 1)	Mean	2011		
	Median	2011		
	Standard Deviation	9.92		
	Min.	1986		
	Max.	2025		

Table S2 - Duration of employment in schools

Duration of school setting working	Frequency	% of Total	% Cumulative
Less than a year	4	3.8%	3.8%
1 year	2	1.9%	5.7%
2-4 years	45	42.9%	48.6%
5-8 years	16	15.2%	63.8%
more than 8 years	38	36.2%	100.0%

Table S3 - Specialist training courses

	Frequency	% of Total	% Cumulative
**BLS or PBLS course**			
No	43	41.0%	41.0%
Yes	62	59.0%	100.0%
**Other courses related to CCC**			
No	84	80.0%	80.0%
Yes	21	20.0%	100.0%
**Coaching and contextualisation with specialised staff at the child's home**			
No	54	51.5%	51.5%
Yes	51	48.6%	100.0%

CCC = Complex Care Children

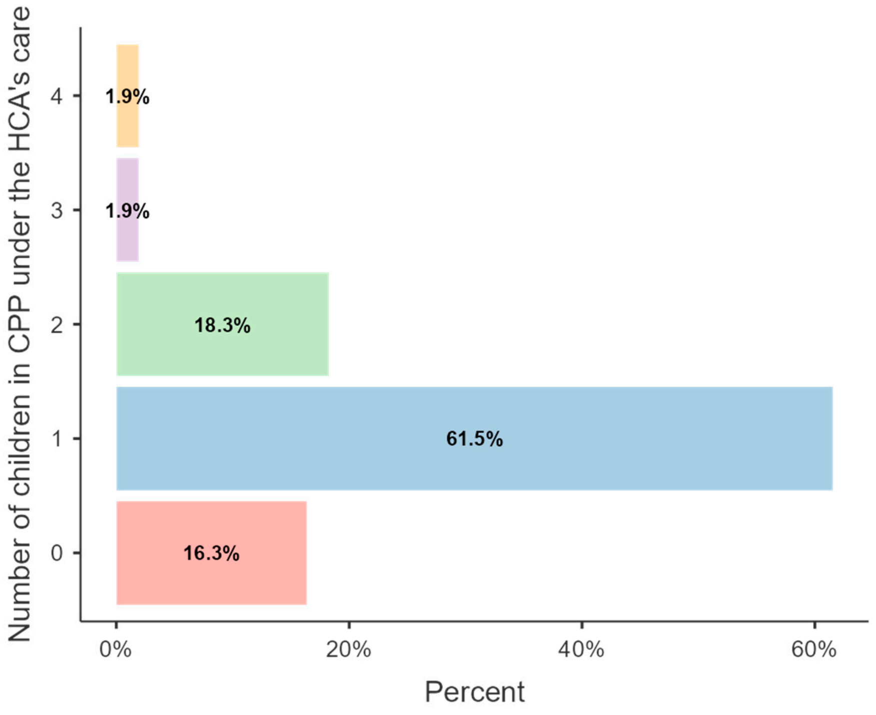

Figure S1 - Number of children in CPP who have been assigned to each HCA since they started working at the school
